# Resuscitation Systems Analysis in Botswana Using the Global Frame of Survival: A Narrative Review

**DOI:** 10.51894/001c.164028

**Published:** 2026-07-31

**Authors:** Michael Goldberg, Mabintou Darboe, Ebtekar Mounzer, Hina Inam, Furqan Irfan

**Affiliations:** 1 College of Osteopathic Medicine, Michigan State University

## 19

### Introduction

Out-of-hospital cardiac arrest (OHCA) survival depends on a coordinated “chain of survival” supported by system factors inside and outside the emergency care system. Botswana has established a government-led emergency medical services (EMS) system and is developing emergency care capacity, but the extent of implementation and system performance across districts remains poorly described.

### Objectives

To map Botswana-specific evidence to the Global Resuscitation Alliance Frame of Survival (outer and inner frames) and summarize the state of system readiness for OHCA, highlighting strengths, gaps, and priority areas for improvement.

### Methods

This study synthesized available evidence and policy documents using the Global Resuscitation Alliance Frame of Survival as the organizing framework. Sources included peer-reviewed studies, government documents, and WHO reports.

### Results

Botswana demonstrates national commitment through the establishment of a government-led EMS function (initiated in 2012), a public access pathway, and emergency preparedness planning. Early operational data from Gaborone (2013-2014) describe EMS activity, supporting documented service delivery at least in the capital region. The policy environment for emergency response exists, but evidence for bystander protections, including Good Samaritan coverage, is limited. Preventive health data show a growing cardiometabolic and chronic disease burden, increasing the likelihood of time-critical emergencies. Within the inner frame, Botswana shows progress in emergency medicine specialty development and facility-level emergency care processes, including triage implementation and time-to-care performance in a district hospital setting. However, hospital resuscitation readiness appears variable in some district hospitals. CPR training and retention needs persist. Rural transfers often rely heavily on nurses and are constrained by staffing and infrastructure limitations. Across multiple elements, performance metrics were sparse, with limited publicly available reporting on response times, OHCA outcomes, bystander CPR, AED access, or registry infrastructure.

### Discussion

Botswana has foundational EMS and facility emergency care components, but performance appears uneven across settings and is limited by major data gaps. Priorities include strengthening equitable EMS coverage and transfer capacity, improving facility resuscitation readiness, building community response, clarifying supportive legal frameworks, and strengthening data systems. Developing national OHCA registries and routine EMS performance monitoring will be critical for guiding policy decisions and evaluating future improvements in resuscitation systems.

